# STING activation in cancer immunotherapy

**DOI:** 10.7150/thno.37574

**Published:** 2019-10-15

**Authors:** Ting Su, Yu Zhang, Kristoffer Valerie, Xiang-Yang Wang, Shuibin Lin, Guizhi Zhu

**Affiliations:** 1 Department of Rehabilitation Medicine, Center for Translational Medicine, Precision Medicine Institute, The First Affiliated Hospital, Sun Yat-sen University, Guangzhou, 510080, China; 2 Department of Pharmaceutics and Center for Pharmaceutical Engineering and Sciences, School of Pharmacy, Richmond, VA, 23298, USA; 3 Massey Cancer Center, Virginia Commonwealth University, Richmond, VA, 23298, USA; 4 Department of Radiation Oncology, Virginia Commonwealth University, Richmond, VA, 23298, USA; 5 Department of Human and Molecular Genetics, Virginia Commonwealth University, Richmond, VA, 23298, USA.; 6 Institute of Molecular Medicine, Virginia Commonwealth University, Richmond, VA, 23298, USA.; 7 Institute for Structural Biology, Drug Discovery and Development, Virginia Commonwealth University, Richmond, VA, 23219, USA

**Keywords:** Stimulator of interferon genes (STING), cyclic dinucleotides, cyclic GMP-AMP synthase (cGAS), immunostimulatory adjuvants, drug delivery, cancer immunotherapy

## Abstract

Cancer immunotherapy modulates and leverages the host immune system to treat cancer. The past decade has witnessed historical advancement of cancer immunotherapy. A myriad of approaches have been explored to elicit or augment anticancer innate immunity and/or adaptive immunity. Recently, activation of stimulator of interferon (IFN) genes (STING), an intracellular receptor residing in the endoplasmic reticulum, has shown great potential to enhance antitumor immunity through the induction of a variety of pro-inflammatory cytokines and chemokines, including type I IFNs. A number of natural and synthetic STING agonists have been discovered or developed, and tested in preclinical models and in the clinic for the immunotherapy of diseases such as cancer and infectious diseases. Cyclic dinucleotides (CDNs), such as cyclic dimeric guanosine monophosphate (c-di-GMP), cyclic dimeric adenosine monophosphate (c-di-AMP), and cyclic GMP-AMP (cGAMP), are a class of STING agonists that can elicit immune responses. However, natural CDNs are hydrophilic small molecules with negative charges and are susceptible to enzymatic degradation, leading to low bioavailability in target tissues yet unwanted toxicities and narrow therapeutic windows. Drug delivery systems, coupled with nucleic acid chemistry, have been exploited to address these challenges. Here, we will discuss the underlying immunological mechanisms and approaches to STING activation, with a focus on the delivery of STING agonists, for cancer immunotherapy.

## 1. Introduction

**Figure 1:**
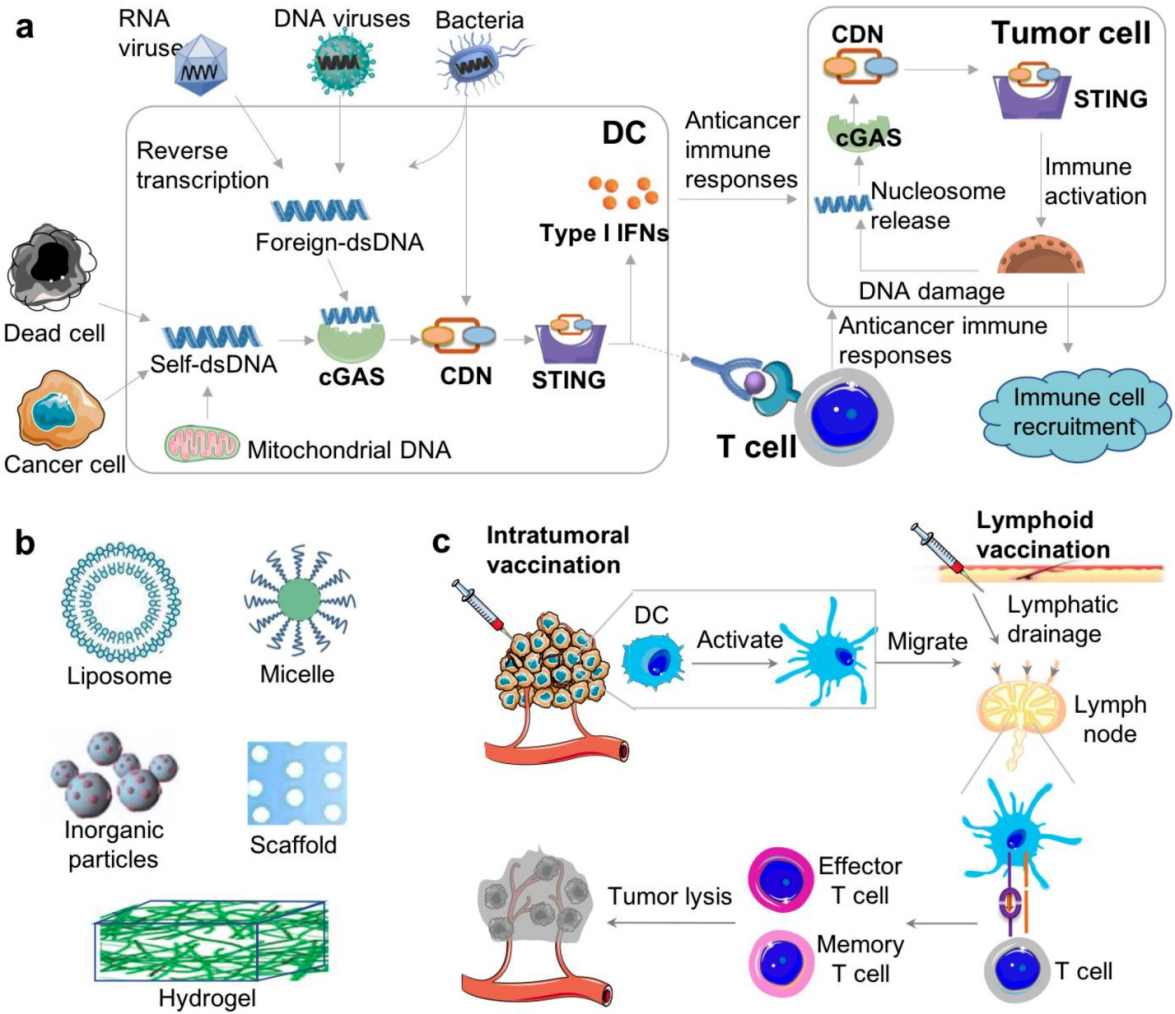
** STING activation for cancer immunotherapy.** (A) The cGAS-STING signaling pathway that mediate the cytosolic nucleic acid sensing and can be activated to elicit antitumor immune responses for cancer immunotherapy. Reprinted from 16, copyright (2017) Elsevier Ltd.. (B) Representative biomaterials that have been exploited to delivery STING agonists, including CDNs. (C) Schematic description of delivering STING agonists, *via* intratumoral vaccination or lymphoid vaccination, to elicit innate and adaptive antitumor immune responses. cGAS: cyclic GMP-AMP synthase; CDN: cyclic di-nucleotide; IFN: interferon; STING: stimulator of interferon genes; DC: dendritic cell; TCR: T cell receptor; MHC-I: major histocompatibility complex type I.

Stimulator of interferon genes (STING) is a signaling molecule that plays a crucial role in controlling the transcription of many host defense genes, including pro-inflammatory cytokines and chemokines, and type I interferons (IFNs) 1, 2. STING appears to be a dimer, with 398 and 378 amino acids in humans and mice, respectively. STING is located on the membrane of endoplasmic reticulum (ER) with its C-terminal tail residing in cell cytosol 3. In early studies, STING was observed to stimulate the transcription of innate immune genes in response to some of invading bacteria, DNA viruses or transfected DNA 1, 2, 4, 5. Further investigation revealed that STING was strongly activated by cyclic dinucleotides (CDNs), such as cyclic di-GMP (c-di-GMP) and cyclic di-AMP (c-di-AMP), both of which can be secreted by bacteria 6, 7. Indeed, cytosolic DNA species can also trigger STING signaling following binding to and activating cyclic GMP-AMP synthase (cGAS). Specifically, in the presence of cytosolic double-stranded DNA (dsDNA), the intracellular nucleic acid sensor cGAS uses cytosolic ATP and GTP as substrates to catalyze the production of cyclic GMP-AMP (cGAMP), which has a noncanonical 2ʹ,5ʹ-phosphodiester linkage and/or a canonical 3ʹ,5ʹ linkage (c[G(2ʹ,5ʹ)pA(3ʹ,5ʹ)p]) 8-10. Upon binding to CDNs, STING translocates from the ER to the Golgi apparatus and further to the perinuclear microsomes or punctuate structures, which in turn recruit the downstream TANK-binding kinase 1 (TBK1) and the transcription factor interferon regulatory factor 3 (IRF3), leading to induction of type I IFNs 11. Typically, STING is then rapidly degraded, an event that may avoid problems associated with sustained cytokine production (Figure 1A) 12. In addition, STING is associated with the sensing of aberrant cytosolic DNA species, including self-ssDNA (single-stranded DNA) and dsDNA, to trigger host-defense-related gene expression 13. Conversely, constitutive STING activation has been linked to autoimmune diseases 14. For example, some gain-of-function mutations in STING result in constitutive activity and autoinflammatory diseases such as STING-associated vasculopathy 15. In this article, we will discuss the underlying immunological mechanisms and approaches to activating STING for cancer immunotherapy, with a focus on potential drug delivery systems for STING agonists (Figure 1).

## 2. cGAS-STING signaling pathway in cancer and cancer immunotherapy

cGAS-STING signaling pathway has the potential to elicit or boost innate and adaptive immune responses, both of which are critical for cancer immunotherapy (Figure 1) 17. The activation of STING drives the production of cytokines such as Type I IFNs 18. Type I IFNs belong to a family of cytokines and consist of 16 members, including 12 IFN-α subtypes, IFN-β, IFN-ε, IFN-κ, and IFN-ω, all of which are involved in antiviral immunity 19. Type I IFNs promote the generation of cytotoxic T cell responses as well as type 1 T helper cell (Th1)-biased responses 20. Furthermore, type I IFNs promote the activation and functional maturation of dendritic cells (DCs), thereby facilitating antigen presentation to CD4^+^ T cells as well as antigen cross-presentation to CD8^+^ T cells 21. STING activation triggers a multifaceted type I IFN-driven inflammatory response that stimulates DC activation and cross-presentation of tumor antigens for the subsequent T cell priming 22. Further, recent studies have shown that the STING signaling pathway is essential for endogenous antitumor T cell responses as well as radiation-induced antitumor T cell responses 23, 24. Consistently, STING-deficient mice have a higher susceptibility to tumor formation, diminished antitumor T cell immunity and impaired responses to immunotherapy 24. Furthermore, the ability of immune checkpoint inhibitors to reinvigorate antitumor immune responses was also abrogated in STING-deficient mice, indicating a role of STING in the therapeutic efficacy of immune checkpoint inhibitors 25. One hypothesis for the underlying mechanism is that DCs engulf necrotic tumor cells, and the tumor cell-derived DNA triggers STING signaling in DCs 23, 24, 26, 27. The resulting type I IFNs, in a paracrine or autocrine manner, may elicit the production of additional cytokines in DCs that facilitate antigen presentation to CD4^+^ T cells and antigen cross-presentation to CD8^+^ T cells, thus further potentiating antitumor T cell responses (Figure 1C).

In addition to T cells, the STING signaling pathway can be activated in macrophages, B cells and some other leukocytes 3, 14 to produce type I IFNs. Moreover, the STING signaling pathway can also be triggered in NK cells, which are then primed for the cytotoxic killing of tumor cells 28. These studies provide the evidences that STING signaling pathway plays a central role in a variety of innate and adaptive immune responses that can be exploited for cancer immunotherapy.

Note that, STING can also be a double-edged sword in cancer development. Cancer cells may resist against the activation of the cGAS-STING pathway. Indeed, low STING signaling activity has been found in multiple types of cancer cells ranging from colorectal carcinoma 29, melanoma 30, to ovarian cancer 31. STING activation can be suppressed often by genetic mutations and/or direct epigenetic silencing of either STING or cGAS. For example, Kirsten rat sarcoma gene (KRAS)- and LKB1-mutated non-small cell lung cancer cells epigenetically silenced STING and cGAS expression 32. Consequently, loss of STING-cGAS signaling rendered these cancer cells unable to elicit antitumor immune responses. Moreover, STING activation has been found to promote the proliferation of brain metastatic cells and chemoresistance in breast cancer cells and lung cancer cells 33. Specifically, brain metastatic cancer cells use astrocyte gap-junctional networks to transfer cGAMP to astrocytes, leading to STING activation in astrocytes and production of inflammatory cytokines. These inflammatory cytokines can activate the STAT1 and NF-κB pathways in brain metastatic cells, thereby supporting tumor growth and chemoresistance. In addition, prolonged IFN-I signaling has been shown to cause immune dysfunction 34. Overall, the potentially opposing functions of STING activation may influence the balance between anticancer immune responses and the immune escape of cancer 35.

## 3. STING-activating drugs

**Figure 2:**
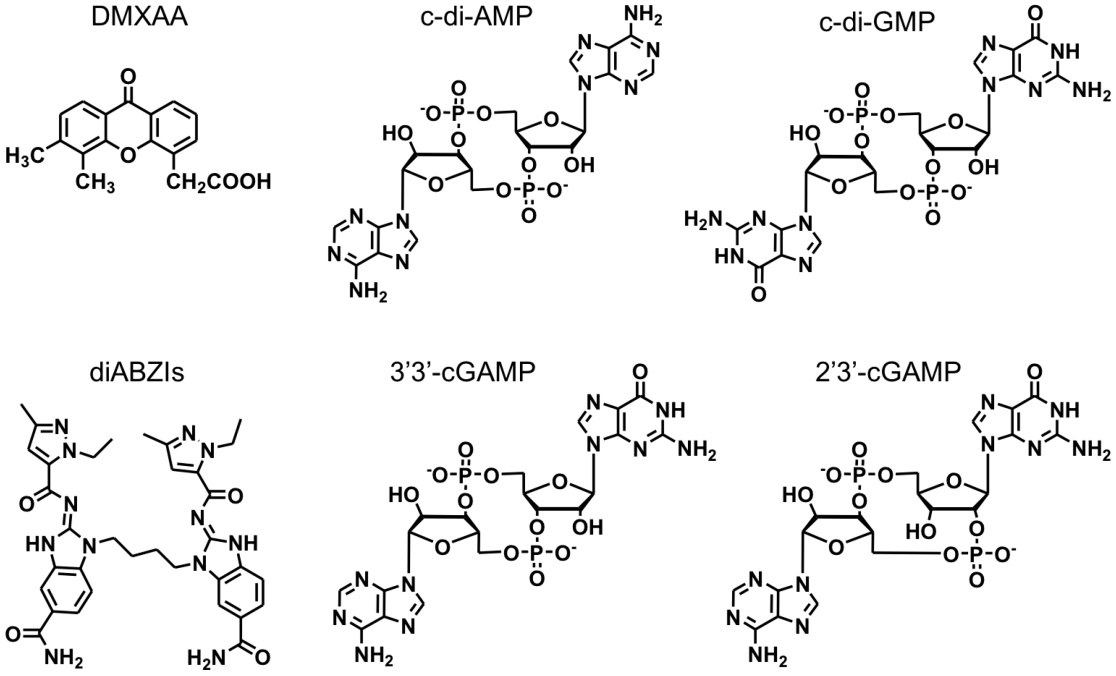
The chemical structures of representative STING agonists.

Insight into the roles of STING in immunomodulation indicated the potential of STING agonists as cancer therapeutics to activate antitumor immune responses 22. Small molecule STING-activating immunomodulators have been long studied for the treatment of diseases, including cancer. An early example of STING activator, 5,6‑dimethylxanthenone‑4‑acetic acid (DMXAA) (Figure 2), was investigated as an experimental anticancer immunomodulator 36. Unfortunately, STING binding and immune activation by DMXAA was only specific for murine STING but not human STING, which is attributed to the unsuccessful clinical translation of DMXAA in human cancer patients 37. Nonetheless, DMXAA has generated tremendous basic knowledge and highlighted the importance of species selectivity in drug development for human diseases. Indeed, small molecule STING-activating immunomodulators are still appealing for the cancer drug development. For example, Ramanjulu, et al reported the discovery of a small molecule STING agonist that is systemically efficacious to treat tumors in mice 38. Specifically, they developed a linking strategy to synergize the effect of two symmetry-related amidobenzimidazole (ABZI)-based compounds to create linked ABZIs (diABZIs) (Figure 2), which was empowered with enhanced STING binding affinity and strong antitumor activity.

CDNs are another type of STING agonists (Figure 2). CDNs that are derived from bacteria might directly activate the STING signaling pathway. Since the late 1980s, CDNs were recognized as secondary messengers that mediate signaling transduction in prokaryotic cells. In mammalian cells, CDNs function as activators of the innate immune responses 39. The potential anticancer activity was discovered in CDNs 40, such as c-di-GMP that inhibited the proliferation of human colon cancer cells *in vitro*. Later, the effect of CDNs on the host immune response was discovered 41-44. When a model antigen β-galactosidase (β-Gal) was administered subcutaneously with c-di-GMP *in vivo*, a significant increase in antigen-specific IgG was observed 45. Cellular immune responses showed that the production of not only IFN-γ, IL-10 and IL-2, but also pro-inflammatory cytokines and chemokines was greatly elevated compared with antigen β-Gal alone 45. By intraperitoneal injection of c-di-GMP, it was found that high-dose c-di-GMP directly killed tumor cells likely *via* inducing immunogenic tumor cell death 46. However, low-dose c-di-GMP improved T-cell responses and significantly reduced immune suppression by converting a subpopulation of immune-suppressing myeloid-derived suppressor cells (MDSCs) into an immune-stimulating phenotype, characterized by the production of IL-12 that can stimulate the activation of T cells 46. One high-dose treatment followed by multiple low doses of c-di-GMP was equally effective compared with the combination of c-di-GMP and Listeria monocytogenes (LM)-based vaccine that expresses a tumor-associated antigen Mage-b (LM-Mb) 46. Increasing evidence indicates that targeting the STING signaling pathway in the tumor can be an important approach to remodeling the tumor microenvironment for immunotherapy 22. 3'3'-cGAMP and 2'3'-cGAMP (Figure 2) are also commonly used CDNs 47, 48. 2'3'-cGAMP is a natural CDN. In different tumor types such as 4T1 murine breast cancer, HSC-2 squamous cell carcinomas, CT26 murine colon cancer, and B16F10 murine melanoma, intratumoral vaccination with 2'3'-cGAMP led to transient accumulation of macrophages at the tumor site and remodel the tumor immune microenvironment by, for example, repolarizing M2-like tumor-associated macrophages to antitumor M1-type macrophages 48. In another study, intraperitoneally-injected 3'3'-cGAMP induced apoptosis in malignant B cells through STING activation 47. Given the ability of STING agonists to elicit potent innate and adaptive immune responses, rational combination of STING agonists with immune checkpoint inhibitors have been explored for cancer immunotherapy. Intratumoral vaccination with STING agonists can potently prime innate immunity and tumor antigen-specific CD8^+^ T cell responses. CDNs increased tumor-specific CD8^+^ T cells infiltration and potentiated the therapeutic efficacy of anti-CTLA-4, anti-PD-1, and anti-4-1BB, and reprogrammed suppressive tumor-associated macrophages to a proinflammatory phenotype, namely M1 macrophage 49. In another example, STING agonists were combined with a PD-L1 inhibitor and an OX40 agonist, resulting in not only effectively activation of innate immunity to support T cell priming, but also overcoming the antigen-enforced immune tolerance for tumor regression 50. These results indicate the great potential of these STING agonists for versatile applications in tumor immunotherapy.

## 4. STING-activating drug delivery systems in cancer immunotherapy

As discussed above, small molecule STING agonists as well as cytosolic DNA species can stimulate the STING signaling pathway to promote antigen presentation and T cell priming for tumor eradication 51-54. However, the intrinsic negative charges, susceptibility to enzymatic degradation, hydrophilicity, as well as small sizes of CDNs pose challenges to the biostability, bioavailability, delivery, and retention of CDNs in target tissues and cells. Drug delivery systems involving biomaterials at a variety of scales (from nanocarriers, microcarriers, to macromaterials) have been engineered to overcome tissue and cell barriers to improve the therapeutic efficacy while ameliorating adverse side effects (Table 1). In general, these delivery systems can be applied under different contexts. Typically, the smaller the drug carriers, the easier for them to be transported via lymphatic drainage which is often need in local vaccination; by comparison, relatively large drug carriers such as large microparticles and hydrogels tend to be retained locally, which may be great for in situ vaccination or intratumoral implantation. Worth noting that, macromaterials such as hydrogels may involve invasive procedure, with the exception of injectable macromaterials. At the tissue level, drug delivery systems have been developed to transport and retain STING agonists in the tissues, such as lymph nodes for lymphoid vaccination, or tumors for intratumoral vaccination. At the cell level, since STING is located in the ER, drug delivery systems are expected to deliver STING agonists across cell membrane and even escape from the endosomal compartment if endocytosis is involved. A variety of such drug delivery systems have been engineered based on nanoparticles 55-58, microparticles 59, 60, as well as macromaterials such as hydrogels 61, 62 (Table 1). In this section, we will discuss the application of STING agonists for cancer immunotherapy, with a focus on drug delivery systems for CDN-based STING agonists.

### 4.1. Nanocarrier-based STING-activating delivery

STING-activating drugs can induce profound antitumor immune responses. However, the clinical translation of CDN-based STING agonists can be confronted by drawbacks of CDNs. First, CDNs are susceptible to enzymatic degradation by phosphodiesterases 73. Second, the hydrophilicity and small sizes (molecular weights lower than 1 kDa) of CDNs facilitate random diffusion and clearance upon typical administration into the body. Third, the negative charges of CDNs refrains CDNs from cell membrane penetration and cell uptake 57, 74. These drawbacks render CDNs to have poor pharmacokinetics and pharmacodynamics, short half-lives, unwanted systemic dissemination that may further cause toxic cytokine storms, and limited bioavailability. Nucleic acid chemistry has been employed to chemically modify CDNs to improve the biostability of CDNs. For instance, one STING agonist called ADU-S100, also known as ML RR-S2 CDA (dithio-(R_P_,R_P_)-[cyclic[A(2',5')pA(3',5')p]), is a phosphodiesterase-resistant CDN that has been tested in the clinic. By chemical modifications, its biostability has been improved which further promotes their antitumor efficacy in a series of cancer cells 75. To address the complications associated with the hydrophilicity and negative charges of CDNs, cationic and/or encapsulating drug carriers have been exploited to improve the tissue and cell delivery of CDNs, while minimizing systemic toxicity 58, 76. Injection of unformulated “free” STING agonists may lead to rapid dissemination into the blood and subsequently cause systemic cytokine storm that can be harmful 77, 78. In one example, c-di-GMP-incorporated nanoparticles elicited 8.2-fold more antigen-specific IgG titers than the “free” c-di-GMP counterpart at the same dose. While elevating the dose of c-di-GMP promoted the production of antibody titer, this is accompanied by the elevated production of systemic inflammatory cytokines such as IL-6, TNF-α, and IFN-β 58. Nanoparticles smaller than 200 nm in diameters can typically be taken up by the peripheral APCs or drained from interstitial spaces to the lymphatic lumen and then transported to draining lymph nodes 79, 80, which host a variety of immune cells and orchestrate immune responses that are critical for cancer immunotherapy. Typically, nanoparticles with diameters of approximately 50 nm have been found to be especially efficient at uptake and retention in lymph nodes 81, 82. Thus, rationally-designed nanoparticulate delivery systems hold tremendous potential to promote CDN delivery and advance the application of CDNs as potent immunotherapeutics 58. A series of CDN delivery systems have been developed using nanocarriers such as liposomes 57, 58, 65, 74, 83, polymeric nanoparticles 55, 59, 68, 84, as well as inorganic materials 72, 85.

Liposomes, which can have positive charges and aqueous cores, are great candidates for encapsulating STING agonists. The positive charge on liposomes can not only promote the encapsulation of negatively-charged CDNs, but can also facilitate intracellular liposome delivery by electrostatically interacting with negatively-charged cell membrane 57, 58, 65, 74, 83. For example, the encapsulation of c-di-GMP in PEGylated lipid nanoparticles concurrently minimized systemic dissemination and markedly enhanced lymph nodes accumulation compared with free c-di-GMP 58. When co-delivered with a peptide antigen, the c-di-GMP-delivering nanoparticles increased antigen-specific CD8^+^ T cell responses. Moreover, the durable antibody titers were substantially higher than those promoted by a TLR4 agonist monophosphoryl lipid A, indicating that the nanoformulation improved the delivery of c-di-GMP and promoted the immune responses of c-di-GMP. This approach implies that the delivery and the cancer therapeutic efficacy of STING agonists such as CDNs can be effectively improved via drug delivery systems based on rationally-designed nanocarriers. Besides functioning as vaccine adjuvants, nanoparticulate STING agonists such as PEGylated liposomes loaded with cGAMP, can also be used to overcome the immunosuppressive tumor microenvironment 74. When intratumorally administered, this liposomal formulation of cGAMP significantly enhanced the tumor retention of cGAMP and the colocalization of cGAMP with tumor-associated APCs, which may explain the superior type I IFN induction and adaptive immune responses to clear established melanoma and to resist a second tumor challenge. Even in PD-L1-insensitive models of triple-negative breast (TNBC) cancer which has poor prognosis and few effective treatment options, STING agonist-loaded liposomes effectively repolarized M2-like macrophages into M1-type macrophages and elicited STING-dependent antitumor immunity 57. Note that, these CDN nanoparticles can elicit a potent and durable immune response that prevents relapse 57, 74.

**Figure 3:**
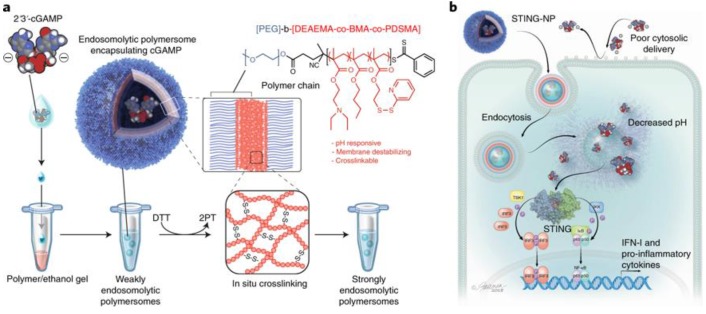
** Polymersome-based CDN delivery for cancer immunotherapy. a)** Schematic illustration of using pH-responsive diblock copolymers to formulate 2′3′-cGAMP-loaded endosomolytic polymersomes. **b)** Schematic description of intracellular uptake of the intracellular delivery of 2′3′-cGAMP via polymersomes (STING-NPs), the endosomal release of 2′3′-cGAMP from STING-NPs, and the endosomal escape of 2′3′-cGAMP to the cytosol for STING activation. Reprinted from 56, copyright (2019) Nature Publishing Group.

Polymeric nanoparticles represent another promising class of nanocarriers for the delivery of STING agonists such as CDNs for caner immunotherapy 55, 56, 59, 68. Polymeric nanoparticles can be tailor-designed with defined topological structures, functional modifications, controlled drug loading and release kinetics, as well as good biodegradability and good safety 81, 84, 86-88. These characteristic features have empowered polymeric nanocarriers to be one of the most successful class of drug nanocarriers. For example, a biodegradable poly(beta-amino ester) (PBAE) cationic polymer was developed to form PBAE/CDN polymeric nanoparticles through electrostatic interaction between positively charged PBAE and negatively charged CDNs 55. The resulting nanoparticles can be effectively and selectively taken up by monocytes and macrophages, indicating the potential of these CDN nanoparticles as immune adjuvants. This selectivity of uptake might be attributed to the end-capping group in the polymers. When combined with an immune checkpoint inhibitor, these CDN nanoparticles showed an order of magnitude reduction of the dose needed to eliminate established poorly immunogenic melanoma. In another example of polymeric nanoparticles for CDN delivery, pH-responsive polymersomes were designed to load cGAMP 69, 70. The pH-responsive polymersomes can disassemble in the acidic endolysosome upon endocytosis-mediated cell uptake, allowing conditional cGAMP release from the polymersome carriers (Figure 3) 56. This polymersome delivery system potentiated the immunostimulatory activity of cGAMP by two to three orders of magnitudes in multiple immune cells *in vitro*. A single-dose intratumoral administration of such CDN-loaded polymersomes in a mouse melanoma model remodeled the tumor immune milieu, as characterized by increased populations of tumor-infiltrating neutrophils as well as CD8^+^ and CD4^+^ T cells, activated DCs indicated by CD86 expression, which altogether reprogram the tumor microenvironment to be 'hot' or T cell-inflamed for efficacious immunotherapy. As a result, when administered intratumorally or intravenously, these cGAMP-loaded polymersomes increased the therapeutic efficacy.

In addition to liposomes and polymeric nanocarriers, some other types of nanocarriers have been investigated to deliver CDNs for cancer immunotherapy. In one example, cationic silica nanoparticles (CSiNPs), which can induce necrotic tumor cell death, were used to further deliver c-di-GMP and elicit strong antitumor immune responses upon intratumoral vaccination 85.In a melanoma mouse model, it was shown that the STING agonists cooperate with the release of tumor-associated antigens and local inflammation in tumor microenvironment induced by the CSiNPs to enhance the immunotherapeutic efficacy.

### 4.2. Micromaterial- or macromaterial-based STING-activating drug delivery

Drug delivery systems based on micromaterials, such as microparticles, have also been developed for STING agonists in cancer immunotherapy. For example, acetylated dextran (Ace-DEX) were developed to synthesize polymeric microparticles for cGAMP loading by electrospray 60. Through a one-step synthesis, the pendant hydroxyl groups of water soluble dextran were converted into acid-sensitive acetal groups. These microparticles demonstrated the potential as a potent vaccine adjuvant to elicit or augment humoral and cellular immune responses including type-I IFN production, antibody production, as well as germinal center B cell and memory T cell responses. Further, the therapeutic efficacy of cGAMP-loaded microparticles was investigated in two murine tumor models. Compared with three clinically-relevant immune-activating drugs (imiquimod, murabutide, and poly(I:C)), intratumorally-administered cGAMP-loaded microparticles generated robust innate and adaptive anti-cancer immune responses and enhanced type-I IFN responses by up to 50 times 59. In another example, the Ace-DEX microparticles were studied for the co-delivery of cGAMP and R848 (a TLR7/8 agonist), and the resulting Ace-DEX microparticles co-loaded with cGAMP and R848 were found to elicit strong immune responses when administered at extremely low doses 68.

Hydrogel-based micromaterials or macromaterials are another interesting class of delivery platform for STING-activating immunomodulators. For example, submicron-sized microparticulate hydrogels were fabricated from linear poly-ethyleneimine (LPEI)/hyaluronic acid (HA), and were loaded with cGAMPs as vaccine adjuvants 62. The resulting microgels mediated efficient intracellular delivery via uptake by phagocytic macrophages, leading to enhanced cytokine induction compared with conventional cationic Lipofectamine. In another example, a peptide hydrogel, called STINGel, was developed as an injectable peptide hydrogel that controllably delivered CDNs. STINGel was formulated through the electrostatic interactions between negatively charged CDNs and the positively charged peptide 61. The controlled release of CDNs from STINGel created a high local CDN concentration that lasted for at least seven days. Such local STING agonist depots around the STINGel can efficiently remodel the tumor immune microenvironment to improve the immunotherapeutic efficacy.

**Figure 4:**
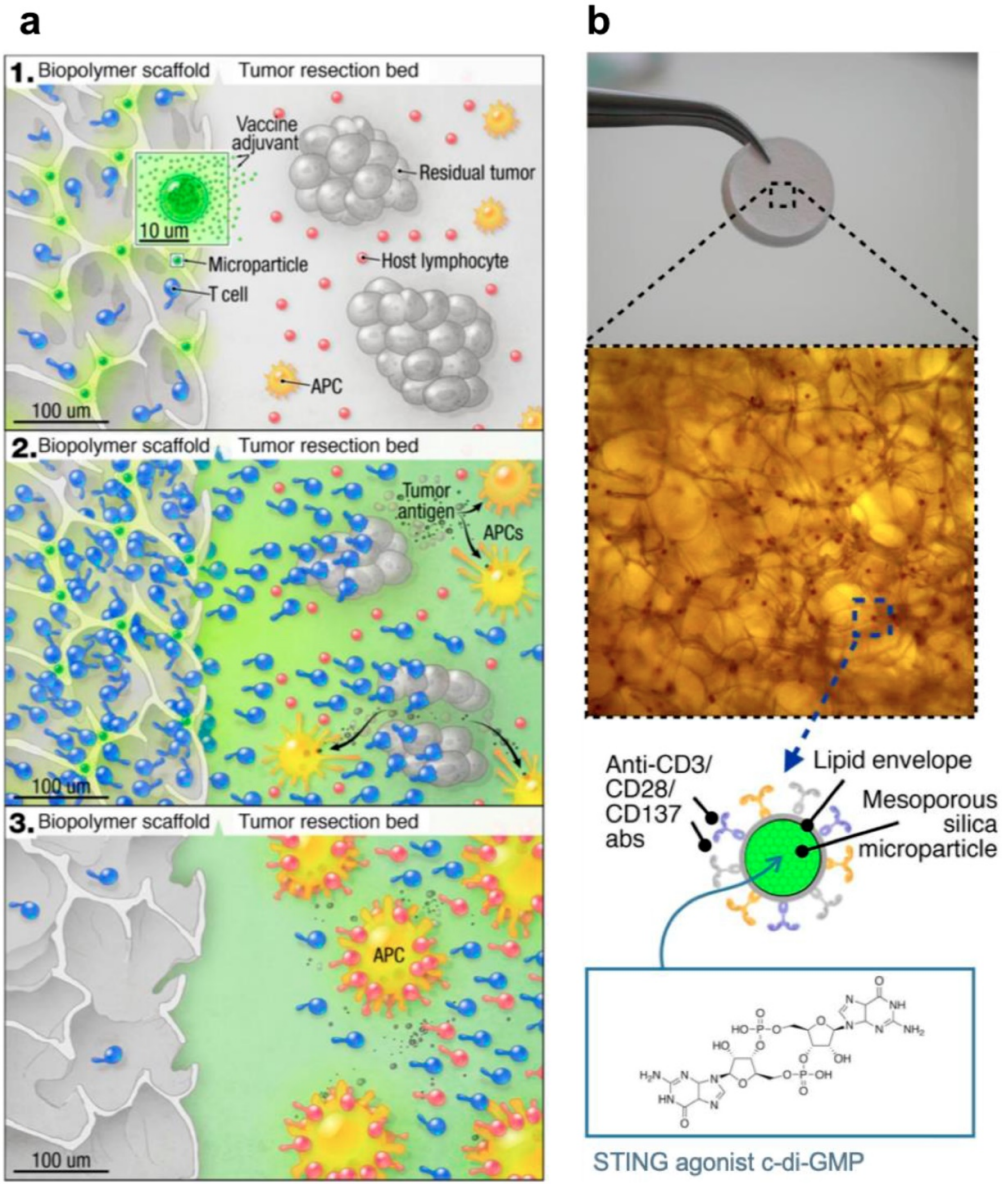
Schematic illustration of an implantable biomaterial scaffold that co-delivered CAR-T cells and CDNs for synergistic tumor immunotherapy. **a)** The scaffold that was loaded with CAR-T cells and microspheres of STING agonists interact with the tumor bed. **b)** The Macro- and microscope image of the porous alginate matrices that are functionalized with c-di-GMP-loading mesoporous silica microparticles. Reprinted from 72, copyright (2017) American Society for Clinical Investigation.

STING-activating drug delivery systems have also been developed to boost the anticancer immune responses in adoptive cell transfer therapy. In one example, a biopolymer scaffold was developed to deliver STING agonists (c-di-GMP) along with chimeric antigen receptor T (CAR-T) cells, and such STING agonist-delivering scaffold was found to prime robust tumor-specific host lymphocyte responses to eliminate local and distant (metastatic) tumors (Figure 4) 72. Specifically, the implantable scaffold of porous alginate matrices were loaded with mesoporous silica microparticles that were loaded with c-di-GMP, and those silica microparticles were further modified with stimulatory anti-CD3/CD28/CD137 antibodies on their phospholipid membrane to facilitate their interaction with CAR-T cells.

In orthotopic tumor models of inoperable pancreatic cancer and incompletely resected melanoma, this scaffold was directly implanted in the tumor tissues, and the scaffold-mediated CAR-T cell delivery induced tumor regression more effectively when compared to systemic CAR-T cell injection. Armed with STING agonists to remodel the tumor immune milieu, this strategy may provide an effective treatment for solid heterogeneous tumors that have poor responses to conventional T cell therapies. Collectively, these results indicate that rationally-designed micromaterials and macromaterials can be developed for efficient delivery of STING agonists as immunostimulatory adjuvants for versatile combination immunotherapy of cancer.

### 4.3. STING-activating nanoparticles

**Figure 5:**
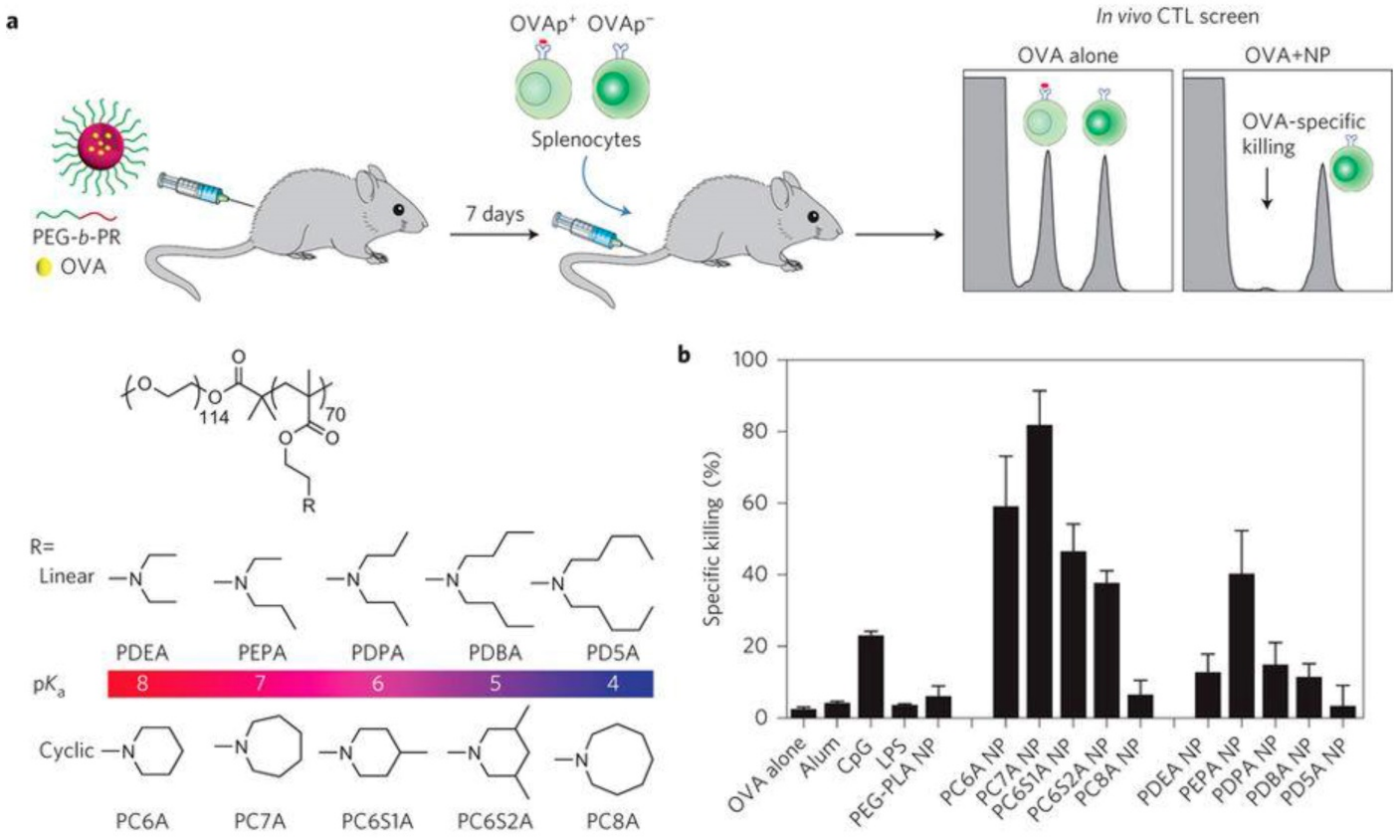
** Intrinsically STING-activating nanoparticles for tumor immunotherapy. a)** Schematic illustration of a series of polymer nanoparticles that were screened for immunostimulation and the generation of strong antigen specific CTL responses when loaded with a model antigen ovalbumin (OVA). **b)** Quantitative comparison of antigen specific CTL responses elicited by different polymer nanoparticles. Reprinted from 69 copyright (2017) Nature Publishing Group.

In addition to serving as carriers for STING agonists, synthetic materials *per se* have also been developed to activate the STING signaling pathway 69, 70. In a recent study, a library of ultra-pH-sensitive copolymers consisting of different tertiary amines was found to activate STING for tumor immunotherapy (Figure 5) 69. The polymers *per se* could activate antigen-presenting cells (APCs), especially DCs in draining lymph nodes and stimulate type I IFN production in a STING-dependent manner. When tumor antigens were delivered *via* these STING-activating nanovaccines, potent and durable antigen-specific T cell responses were elicited, which resulted in robust immunotherapeutic efficacy in multiple murine cancer models. The unique STING activation characteristics of these polymers indicate their potential for application in cancer immunotherapy. In a follow-up study, STING-activating nanoparticles were combined with ionizing radiation 70. This combination of STING-activating nanovaccine with local radiotherapy reverted the immunosuppressive environment in a STING-dependent manner, leading to synergistic radioimmunotherapy in both primary and metastatic tumors.

## 5. Indirect STING-activating therapy

Radiation or some chemotherapeutic drugs can induce immunogenic cell death (ICD). Tumor cell ICD may further induce innate and adaptive antitumor immune responses 89, 90. For example, irradiation was found to induce the production of type I IFNs and elicit adaptive immune responses to support tumor regression 91. Among a variety of mechanisms that could induce type I IFN production, Weichselbaum and co-workers found that STING, but not myeloid differentiation primary-response protein 88 (MyD88) or TIR-domain-containing adapter-inducing interferon-β (TRIF), is indispensable for the induction of type I IFNs and promotion of the antitumor effect of radiation 52. The mechanism of radiation-induced immunostimulation is dose-dependent. When radiation was delivered at a high dose, the induction of three prime repair exonuclease 1 (Trex1) in irradiated cancer cells can degrade the DNA accumulating in the cytosol, which precluded the activation of cGAS-STING-IFN-I pathway and dampens the radiation-induced immunostimulation. By contrast, when radiation was given below the threshold dose for Trex1 induction, cancers cells can be optimally stimulated to produce IFNβ and activate specific DCs, which was essential for the priming of tumor-specific CD8^+^ T cells 92. These studies have provided new insight as to the design of radiotherapy or radioimmunotherapy for the optimal treatment outcome.

In addition to radiotherapy, some chemotherapeutic antitumor drugs that interfere with genomic DNA synthesis or induce genomic DNA damage may induce the production of cytosolic DNA, which trigger cGAS-STING signaling pathway and subsequently elicit antitumor immune responses 93, 94. For example, Topotecan (TPT) can inhibit topoisomerases and trigger DNA double-strand breaks to cause cell death.^68^ This process induces the generation of danger-associated molecules, triggers DC activation, and activates a STING-dependent pathway for antitumor cytokine production. Notably, the antitumor effects of TPT decreased in STING-deficient mice, suggesting that type I IFN production was induced through the cGAS-STING axis and that cGAS-STING axis may play important roles in TPT-induced therapeutic efficacy 95. In another example, hydroxyurea and cisplatin were shown to cause DNA damage in BRCA1-deficient breast tumors, which upregulated the secretion of C-X-C motif chemokine 10 (CXCL10) and chemokine (C-C motif) ligand 5 (CCL5) chemokine in a DNA damage-associated manner involving a STING-TBK1-IRF3 signaling pathway 96. Recently, a poly(ADP-ribose) polymerase (PARP) inhibitor Olaparib was shown to trigger robust STING-dependent antitumor immune responses in breast cancer type 1 (BRCA1)-deficient ovarian cancer, which induces robust adaptive and innate antitumor immune responses. These results shed lights on the mechanisms of the therapeutic effects of PARP inhibitors in BRCA1-deficient tumors 97. With accumulating evidence supporting that the effects of chemotherapeutic drugs involve immunostimulatory pathways such as cGAS-STING, it is expected that the delineation of the immune-related signaling pathways will help map out the comprehensive mechanisms of action for these drugs, and guide the rational drug combinations for cancer therapy.

The STING-dependent antitumor immune responses may mediate the therapeutic activity of oncolytic viruses. As biological nanoparticles that have tumor tropism, oncolytic viruses can target multiple steps in the cancer-immunity cycle 98. These viruses can lyse tumor cells, release tumor antigens (e.g., neoantigens) and danger signals as well as proinflammatory factors such as type I INFs, all of which drive antitumor immune responses. Engineered oncolytic viruses may additionally express cancer therapeutics of interest to drive antitumor immune responses and remodel the tumor immune microenvironment. Following viral infection, viruses can be recognized by pattern recognition receptors (PRRs), such as cGAS and STING. cGAS-STING signaling pathway can sense the genomic elements of viruses, thereby triggering the expression of type I IFNs, the release of chemokines to recruit lymphoid cells that can be leveraged for tumor therapy 98-100.

## 6. Summary and outlook

The cGAS-STING signaling pathway is a critical process in immune sensing that results in the production of type I IFNs, pro-inflammatory cytokines, and chemokines. The characteristic features of STING activation enable STING to be a potential target for cancer immunotherapy, and STING agonists have been investigated for cancer immunotherapy. Optimal cancer immunotherapeutic efficacy is hinged on the effective delivery of such STING agonists to the desired tissue and cell populations. Specifically, a variety of cell populations in tumor and/or immune tissues such as lymph nodes and spleens have shown the potential as targets for STING activation in cancer immunotherapy. Therefore, at the tissue level, drug delivery systems that enable efficient delivery of STING agonists to tumor and/or lymph nodes have been enthusiastically explored. Given the intracellular location of STING in the ER, STING agonists are expected to penetrate cell membrane to interact with STING. CDNs and CDN derivatives are a representative class of small-molecule STING agonists. Natural CDNs are hydrophilic, negatively charged, and are susceptible to enzymatic degradation, all of which present challenges for the tissue and cell delivery of CDNs in cancer immunotherapy. To address these complications, a variety of CDN delivery systems have been developed to improve their efficacy of cancer immunotherapy. In this article, we have summarized recent progress in the development of biomaterial-based STING agonist delivery systems using nanoparticles or microparticles as well as hydrogels. The therapeutic benefit of these delivery systems in preclinical tumor models has been thus far encouraging and insightful for their clinical translation. Worth noting, STING activation appears to be amenable for versatile evidence-based combination with synergistic therapeutics to further improve cancer therapeutic efficacy. Combinations of STING agonists with immune checkpoint blockade has been under clinical investigation for cancer immunotherapy. Moreover, accumulating evidence suggests that STING activation may be involved, largely *via* intracellular nucleic acid sensing, in the process of apoptosis, pyroptosis, necroptosis, and autophagy 101. Given the complexity of the immune modulation network, caution has to be taken in the design of STING-activation-based combination therapy to improve therapeutic outcome while improving or at least not compromising the safety profiles of treatment. Comprehensive delineation of the underlying mechanisms and systematic optimization of immunotherapy involving STING-activating modalities will be intriguing for the design and development of rational combination treatment. Overall, STING activation has shown tremendous potential for cancer immunotherapy, and drug delivery systems can further promote the efficacy of combination cancer immunotherapy.

## Figures and Tables

**Table 1:** STING-activating delivery systems for cancer immunotherapies.

Nanocarriers		Payload CDNs	Tumor models	Administration routes	References
Liposome	PEG-containing lipids	2'3'-cGAMP	Melanoma	Intratumoral	63
	A pH-sensitive cationic lipid (YSK05)	c-di-GMP	Lung metastatic melanoma	Intravenous	64
	A pH-sensitive cationic lipid (YSK05)	c-di-GMP	T cell lymphoma	Subcutaneous	65
	PEGylated lipid	c-di-GMP	Lymphoma; Melanoma	Subcutaneous	58
	Soy-PC-DOTAP liposome	3'3'-cGAMP	Basal-like triple-negative breast cancers; melanoma	Intravenous	57
Polymeric nanoparticles	Poly(beta-amino ester) (PBAE)	ML-RR-CDA	Melanoma	Intratumoral	66
	In situ crosslinked PEG- DBP polymersomes	2'3'-cGAMP	Melanoma	Intratumoral; intravenous	56
	Acetalated dextran (Ace-DEX) microparticles	3'3'-cGAMP	Melanoma	Intraperitoneal; intramuscular; intravenous; intratumoral	59,67,68
	Ultra-pH-sensitive copolymers	--	Melanoma	Subcutaneous	69,70
Others	Cationic silica nanoparticles (CSiNPs)	c-di-GMP	Melanoma	Intratumoral	85
	Irradiated GM-CSF-secreting whole-cell vaccine	CDN derivative	Melanoma	Subcutaneous	71
	Lipid-coated silica microsphere	c-di-GMP	Pancreatic cancer	Implants	72
	LPEI/HA hydrogels	cGAMP	--	Intratumoral	62
	Peptide STINGel	ML RR-S2 CDA	Oral cancer cell	Intratumoral	61

GM-CSF: granulocyte-macrophage colony-stimulating factor; PEG-DBP: poly(ethylene glycol)-block-[(2-(diethylamino)ethyl methacrylate)-co-(butyl methacrylate)-co-(pyridyl disulfide ethyl methacrylate)] copolymers. LPEI: linear poly-ethyleneimine; HA: hyaluronic acid.
